# How genetic variation is affected by geographic environments and ploidy level in *Erianthus arundinaceus*?

**DOI:** 10.1371/journal.pone.0178451

**Published:** 2017-05-30

**Authors:** Jianbo Zhang, Jiajun Yan, Xiaoyun Shen, Dan Chang, Shiqie Bai, Yu Zhang, Jin Zhang

**Affiliations:** 1 Sichuan Academy of Grassland Science, Chengdu, Sichuan, China; 2 State Engineerting Technology Institute for Karst Desertification Control, Guizhou Normal University, Guiyang, China; 3 School of Life Science and Engineering, Southwest Universtiy of Science and Technology, Mianyang, China; 4 Foreign capital project management center, Guizhou proverty alleviation and development office, Guiyang, China; Tulane University Health Sciences Center, UNITED STATES

## Abstract

*Erianthus arundinaceus* is not only a candidate plant for sugarcane breeding programs, but also a potential bioenergy grass. Genetic variation that is affected by geographic environments and ploidy level is very important for the utilization of *Erianthus arundinaceus*. In this study, effects of geographic environments and ploidy level on genetic variation were studied through analyzing the genetic diversity, genetic similarity and cluster analysis of 46 *E*. *arundinaceus* materials from natural habitats in China by using 7 ISSRs and 15 SSRs. Results showed that: 1) Seven ISSRs generated total 66 bands, of which 77% were polymorphic bands, the Nei's genetic similarity coefficient of tested materials ranged from 0.642 to 0.904 with an average value of 0.765. Fifteen SSRs generated 138 bands, of which 81% were polymorphic bands, the Nei's genetic similarity coefficient of tested materials ranged from 0.634 to 0.963 with an average value of 0.802. The results indicated great genetic diversity existed in the tested materials. 2)The tested materials were clustered into 3 groups and 7 subgroups, which demonstrated a strong geographic effect on variation of the local *E*. *arundinaceus*, and weak relationship was found between genetic distance and geographic distance. Five tetraploid materials were not clustered together, and were clustered together with materials from similar geographical location. 3) The genetic variation and cluster results were affected by geographic landforms and environments, the gene flow was blocked by Ocean and mountains, and promoted by river. The effect of ploidy level on genetic variation was little.

## Introduction

*E*. *arundinaceus* is a warm-season, and caespitose perennial tall grass in *Erianthus* (Poaceae) native to China which are widely distributed in south China [1]. And it is also widely distributed in other south and southeast Asian regions [2]. Tetraploid (2n = 4x = 40) and hexaploid (2n = 6x = 60) are the two major cytotypes in *E*. *arundinaceus*[3]. *E*. *arundinaceus* was onsidered to be a member of the “sugarcane complex” due to its relation with taxa in *Saccharum*, *Erianthus*, *Sclerostachya* and *Narenga* [4]. Early studies of *E*. *arundinaceus* were focused on sugarcane breeding [5] to improve the traits like disease resistance and tolerance to abiotic stresses [6]. Although it is not easy to cross the two species of *Saccharum* and *Erianthus*, hybrids of *Saccharum* × *Erianthus* were successfully generated from some breeding projects [7–10]. Recently, the species has been targeted as a bioenergy perennial for its high fiber, high tillering ability and biomass yield potential on marginal lands [11].

Information of genetic diversity is important for studying the genetics of *E*. *arundinaceus*, and breeding new cultivars in the species. Morphological traits were studied as indicators in genetic diversity of *E*. *arundinaceus*, and high level genetic diversity of *E*. *arundinaceus* from China and low level genetic diversity of *E*. *arundinaceus* from Indonesia were found from previous studies [12,13]. Genetic diversity of *E*. *arundinaceus* has been well documented by using DNA markers in the last two decades. Markers like AFLP, ISSR, RAPD and RFLP used in characterizing genetic diversity in *E*. *arundinaceus* showed high genetic variation existing among these materials of China [14–18]. However, these reports revealed very limited information on genetic variation and laws of genetic variation affected by geographical environment and ploidy level, which could help better understanding the natural variation in the species on a large geographic scale.

In this study, 45 *E*. *arundinaceus* materials from natural habitats in China were studied, in which, 5 materials were tetraploid and other materials were hexaploid. Genetic diversity, genetic similarity and cluster analysis of 45 *E*. *arundinaceus* materials were analyzed by using ISSR and SSR markers, and the effect of geographic environments and ploidy level on genetic variation of *E*. *arundinaceus* will be revealed.

## Materials and methods

### Ethics statement

This study was approved by Sichuan Academy of Grassland Science, Guizhou Normal University, Southwest University of Science and Technology, Guizhou proverty alleviation and development office. No specific permissions were required for collecting *Erianthus arundinaceus* samples at the locations in China, because the research was funded by the National Natural Science Foundation of China, the earmarked fund for China Agriculture Research System, the state key research and development program in the 13th five-year.

### Plant materials and DNA extraction

Materials in this study were collected from 46 wild *E*. *arundinaceus* populations covering Shanxi, Sichuan, Yunnan, Guizhou, Guangxi, Guangdong and Hainan of China. Each sampled was composed of 10 individuals in each population. DNA was extracted from the mixed leaves of each sample. Two cytotypes constituted the materials, of which 5 materials were tetraploid whereas 41 materials were hexaploidy (Table 1) [19]. DNA was extracted from fresh leaves tissue of 4-week plants by using the modified CTAB method of Doyle [20]. Purity and concentration of the genomic DNA were determined by a Nanodrop spectrophotometer (NanoDrop Products, Wilmington, DE). Finally, DNA samples were diluted to 25 ng/μL, and stored at -20°C.

**Table 1:** Description of the plant material studied.

No.	Origin	Altitude(m)	Latitude(N.)	Longitude(E.)	Habitat	Ploidy
SAG-E1	Hanzhong, Shanxi	546.2	33°18′	106°96′	Roadside	Hexaploid
SAG-E2	Guangyuan, Sichuan	642	32°27′	105°48′	Roadside	Hexaploid
SAG-E3	Suining, Sichuan	310	30°32′	105°32′	Hillside	Hexaploid
SAG-E4	Wenjiang, Sichuan	396	29°38′	103°40′	Roadside	Hexaploid
SAG-E5	Shuangliu, Sichuan	550	30°24′	103°54′	Roadside	Hexaploid
SAG-E6	Longquan, Sichuan	709	30°33′	104°18′	Hillside	Hexaploid
SAG-E7	Dujiangyan, Sichuan	725	31°1′	103°35′	Riverside	Hexaploid
SAG-E8	Xinjin, Sichuan	515	30°23′	103°48′	Hillside	Hexaploid
SAG-E9	Jintang, Sichuan	436	30°44′	104°29′	Hillside	Hexaploid
SAG-E10	Jiajiang, Sichuan	426	29°47′	103°41′	Roadside	Tetraploid
SAG-E11	Leshan, Sichuan	362	29°35′	103°46′	Riverside	Hexaploid
SAG-E12	Meishan, Sichuan	408	30°2′	103°50′	Riverside	Hexaploid
SAG-E13	Ya′an, Sichuan	584	29°58′	102°16′5	Riverside	Hexaploid
SAG-E14	Shimian, Sichuan	1024	29°27′	102°11′	Hillside	Hexaploid
SAG-E15	Hanyuan, Sichuan	891	29°26′	102°37′	Roadside	Hexaploid
SAG-E16	Panzhihua, Sichuan	962	26°37′	101°48′	Riverside	Hexaploid
SAG-E17	Huili, Sichuan	1743	26°38′	102°15′	Hillside	Hexaploid
SAG-E18	Ningnan, Sichuan	694	26°58′	102°48′	Riverside	Hexaploid
SAG-E19	Mengma, Yunnan	531	30°23′	099°48′	Roadside	Tetraploid
SAG-E20	Shuangjiang, Yunnan	887	29°32′	099°49′	Hillside	Tetraploid
SAG-E21	Lanchang, Yunnan	436	30°44′	099°48′	Hillside	Hexaploid
SAG-E22	Meng′an, Yunnan	546	30°40′	099°20′	Hillside	Tetraploid
SAG-E23	Duyun, Guizhou	840.8	26°16′	107°29′	Hillside	Hexaploid
SAG-E24	Dushan,Guizhou	943.8	25°45′	107°34′	Riverside	Hexaploid
SAG-E25	Libo, Guizhou	546.3	25°27′	107°53′	Roadside	Hexaploid
SAG-E26	Sandu, Guizhou	740.8	25°30′	107°31′	Roadside	Hexaploid
SAG-E27	Congjiang, Guizhou	180	25°47′	109°3′	Riverside	Hexaploid
SAG-E28	Rongjiang, Guizhou	235	25°56′	108°31′	Riverside	Hexaploid
SAG-E29	Nandan, Guangxi	1127	25°6′	107°29′	Riverside	Hexaploid
SAG-E30	Wuzhou, Guangxi	39	23°29′	111°15′	Wasteland	Hexaploid
SAG-E31	Nanning, Guangxi	89.7	22°37′	108°23′	Hillside	Hexaploid
SAG-E32	Zhongshan, Guangxi	157.6	24°27′	111°5′	Wasteland	Hexaploid
SAG-E33	Guilin, Guangxi	166	25°18′	110°8′	Shrub slope	Hexaploid
SAG-E34	Sanjiang, Guangxi	168	25°46′	109°38′	Riverside	Hexaploid
SAG-E35	Gaozhou, Guangdong	40	21°53′	110°50′	Roadside	Tetraploid
SAG-E36	Shuixi, Guangdong	43	21°33′	110°00′	Roadside	Hexaploid
SAG-E37	Leizhou, Guangdong	38	21°20′	110°13′	Roadside	Hexaploid
SAG-E38	Xuwen, Guangdong	9	20°55′	110°03′	Field edge	Hexaploid
SAG-E39	Xinyi, Guangdong	109.4	22°20′	110°54′	Roadside	Hexaploid
SAG-E40	Anding, Hainan	40	19°05′	110°10′	Riverside	Hexaploid
SAG-E41	Dunchang, Hainan	140	19°12′	109°59′	Wasteland	Hexaploid
SAG-E42	Wuzhishan, Hainan	214	18°59′	109°33′	Hillside	Hexaploid
SAG-E43	Baoting, Hainan	71	18°33′	109°37′	Field edge	Hexaploid
SAG-E44	Dongfang, Hainan	-2	18°56′	108°41′	Wasteland	Hexaploid
SAG-E45	Sanya, Hainan	30	18°33′	109°37′	Wasteland	Hexaploid
SAG-E46	Changjiang, Hainan	78	19°19′	108°58′	Wasteland	Hexaploid

### ISSR reactions

Seven ISSR primers were synthesized by Shanghai Biochemical Engineering Technology (Shanghai, China) (Table 2). PCR reactions were performed in a total volume of 20 μL reactions containing 1μL DNA, 12.5 μL 2× Reaction Mix Which contained 500 μM dNTP each, 20 mM Tris-HCl (pH8.3), 100 mM KCl and 3 mM MgCl_2_ (Tiangen Beijing, China), 0.2 μL (units) Golden DNA Polymerase (Tiangen Beijing, China), 1 μL 10 mM primer, and 5.3 μL of sterile water. PCR amplification reactions were performed in a Mastercycler Pro (Eppendorf, Germany) under the following thermal conditions: 7 min at 94°C; 45 cycles of 94°C for 30 sec, 52°C for 45 sec, and 72°C for 2 min; extension of 7 min at 72°C; and a final storage at 4°C. Products of PCR reactions were separated using 2% agarose gel (contains 4μL/100ml Gelred). After electrophoresis, the gel was observed using Gel Doc(TM) XR System (Bio-Rad, USA).

**Table 2:** Characteristics of ISSR and SSR markers.

	Primer (pairs)	Sequence (5'-3')	Repeat motif	Ta(°C)	Size(bp)	GenBank Accession no.
	808	AGAGAGAGAGAGAGAGC	-	-	-	-
	813	CTCTCTCTCTCTCTCTT	-	-	-	-
	834	AGAGAGAGAGAGAGAGYT	-	-	-	-
	840	GAGAGAGAGAGAGAGAYT	-	-	-	-
	847	CACACACACACACACARC	-	-	-	-
	849	GTGTGTGTGTGTGTGTYA	-	-	-	-
ISSR	892	TAGATCTGATATCTGAATTCCC	-	-	-	-
	BML4	f: AGGGACAAGGCGAGCAAG	(TG)7	58	175–190	KU161120
		r: CAGCATCGGAGTACACAGCA				
	BML8	f: ACGTACGAGTGCTTTTCCG	(AC)10	56	209–230	KU161121
		r: GAAGCCTCGCTAATAAAGTCAG				
	BML9	f: AGCTCTATCGGCTGTTGGGT	(AG)21	59	141–176	KU161122
		r: TGATTGGCTTCTCATTGGGAC				
	BML11	f: ACAAATCTGTGAGACTGTGACCG	(AG)21	59	141–178	KU161123
		r: GGTGCGAATCATGAGGACAG				
	BML12	f: ACTTTGCTTAAAGGAGTTCATCAAG	(AG)12	59	157–184	KU161124
		r: AGGGTGGCAAAAATGGGCT				
	BML16	f: TGATTGATTGGCTCCCCTC	(GA)9	58	138–165	KU161125
		r: AGGCAGAGTTTCTCCAGGCT				
	BML19	f: AGGCTATAACCACCGGAACA	(CT)12T(TC)5	57	187–223	KU161126
		r: ATTGCACGGTCCAGAGAGAC				
	BML21	f: CTCTGCTGAACCAGCGAAGA	(TC)9	59	117–135	KU161127
		r: ATTTGGGTCACAAATACACTTGC				
	BML23	f: CACACACTCACGCGCACAG	(TC)25(AC)7	59	87–133	KU161113
		r: ACCTGCCAAGGTACATCACG				
	BML27	f: ACGTCACCTTCAGCAGCG	(AG)13	58	203–230	KU161114
		r: AGTCCTCATTTCCTCAACGTCT				
	BML30	f: ATGACGGGGCAAGCAGAG	(GA)16	59	141–167	KU161115
		r: ACGGTTACTATGGGACCACATCT				
	BML35	f: AACGGCGCGACAAAACT	(GA)9(GA)6	57	122–143	KU161116
		r: AGGCCCACCTCTTTTAATTCT				
	BML39	f: AAATACACTTGCCCATTGGC	(AG)10	58	113–129	KU161117
		r: ATCTGCTCTGCTGAACCAGC				
	BML45	f: AGACAATATCGGTCGTTACGC	(AG)16	58	156–190	KU161118
		r: AGTGACCACTGCCCAGTGTG				
	BML49	f: AGCTCTTCTTTCCGCGTTTC	(GA)17	59	118–149	KU161119
SSR		r: CATGTGGCGCAACTCCTC				

### SSR reactions

Fifteen SSR primer pairs were synthesized by Shanghai Biochemical Engineering Technology (Shanghai, China) (Table 2). PCR reactions were performed in a volume of 20 μL reactions containing 1μL DNA, 12.5 μL 2× Reaction Mix Which contained 500 μM dNTP each, 20 mM Tris-HCl (pH8.3), 100 mM KCl and 3 mM MgCl_2_ (Tiangen Beijing, China), 0.2 μL (units) Golden DNA Polymerase (Tiangen Beijing, China), 1 μL 10 mM forward primer, 1 μL 10 mM reverse primer, and 4.3 μL of sterile water. PCR amplification reactions were performed in a Mastercycler Pro (Eppendorf, Germany) under the following thermal conditions: 5 min at 95°C; 30 cycles of 94°C for 30 sec, Tm for 45 sec, and 72°C for 45 sec; 10 cycles of 94°C for 30 sec, 53°C for 45 sec, and 72°C for 45 sec; extension of 12 min at 72°C; and a final storage at 4°C. Products in PCR reactions were separated using 5% denatured polyacrylamide gels [acrylamide-bisacrylamide (19:1), 1.0×TBE]. After electrophoresis, the gel was stained in AgNO_3_ solution. The gel image was then photographed by Gel Doc(TM) XR System (Bio-Rad, USA).

### Data analysis

Clearly amplified PCR bands were visually scored for presence as “1” or absence as “0” and input into an Excel matrix for the following analysis. Total bands, polymorphic bands and percent polymorphic bands were figured out respectively. A UPGMA tree based on Nei’s [21] genetic distance data was generated by FreeTree program [22] to examine genetic relationships of materials, bootstrap values were generated by using FreeTree program [22] based on resampling replacements over loci in 1000 replicates. In addition, a mantel test was conducted to calculate the correlation between pairwise geographic and Nei’s genetic distances by using NTSYS software [23]. The computation of the Spearman rank correlation between SSR gene diversity (HE) values and ISSR HE values was carried out by using SAS 8.0.

## Results

### Diversity of ISSR and SSR amplified markers

Seven ISSR markers which produced clear and previous bands were screened out from 100 random markers. A total of 66 bands were obtained through these 7 markers after amplification, of which 51 bands were polymorphic (S1 Table). For each marker, the bands number ranged from 6 to 14, while the number of polymorphic bands varied from 3 to 12. The average number of amplified bands was 9.4, and the average number of polymorphic bands was 7.3. The percentage of polymorphic bands (PPB) ranged from 50% (847) to 88% (813) with an average of 77% (Table 3).

**Table 3:** ISSR and SSR primers sequences and amplified result.

	Primer pair(s)	Total bands(TNB)	Polymorphic bands(NPB)	Percent polymorphic bands(PPB)
	808	14	12	86%
	813	8	7	88%
	834	12	10	83%
	840	13	10	77%
	847	6	3	50%
	849	6	5	83%
	892	7	4	57%
	Mean	9.4	7.3	77%
ISSR	Total	66	51	
	BML4	13	10	77%
	BML8	16	13	81%
	BML9	8	8	100%
	BML11	5	5	100%
	BML12	7	5	71%
	BML16	9	6	67%
	BML19	12	11	92%
	BML21	7	4	57%
	BML23	9	7	78%
	BML27	11	9	82%
	BML30	7	6	86%
	BML35	12	10	83%
	BML39	6	5	83%
	BML45	9	7	78%
	BML49	7	6	86%
	mean	9.2	7.5	81%
SSR	Total	138	112	

A total of 138 bands were obtained by 15 SSR markers from amplification, of which 112 bands were polymorphic (S2 Table). Five to sixteen bands (four to thirteen polymorphic bands) were produced by each SSR marker, of which the average number of amplified bands was 9.2, and the average for polymorphic bands was 7.5. The percentage of polymorphic bands (PPB) ranged from 57% (BML21) to 100% (BML9 and BML11) with an average of 81% (Table 3).

### Genetic similarity analysis of *E*. *arundinaceus*

For ISSRs, values of genetic similarity (GS) ranged from 0.634 to 0.963 with an average of 0.802 among the 46 materials, 0.634 to 0.963 with an average of 0.802 in the materials of Sichuan, 0.835 to 0.902 with an average of 0.878 for Yunnan materials, 0.795 to 0.907 with an average of 0.849 for Guizhou materials, 0.833 to 0.963 with an average of 0.887 for Guangxi materials, 0.841 to 0.962 with an average of 0.865 for Guangdong materials, and 0.892 to 0.986 with an average of 0.865 for Hainan materials, respectively (S3 Table).

For SSRs, values of GS ranged from 0.642 to 0.904 with an average of 0.765 in the 46 materials, 0.744 to 0.907 with an average of 0.821 for the materials from Sichuan, 0.776 to 0.912 with an average of 0.828 for Yunnan materials, 0.807 to 0.877 with an average of 0.851 for Guizhou materials, 0.807 to 0.896 with an average of 0.849 for Guangxi materials, 0.805 to 0.904 with an average of 0.859 for Guangdong materials, and 0.807 to 0.915 with an average of 0.872 for Hainan materials, respectively (S4 Table).

### Cluster analysis

**Fig 1:**
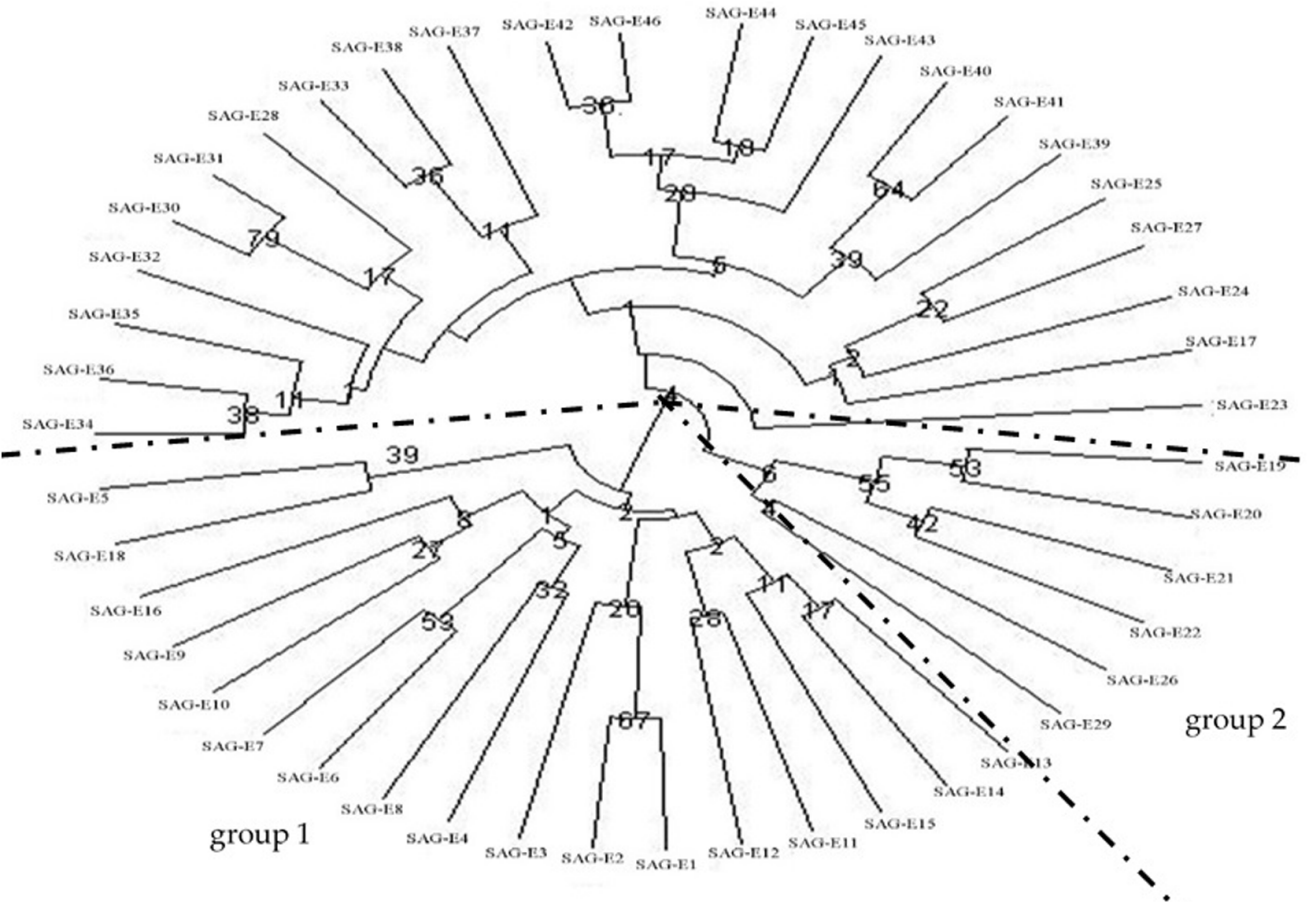
UPGMA cluster analysis based on Nei’s genetic distances of ISSR.

For ISSRs, the UPGMA tree was constructed based on the values of genetic distance. Total 46 materials were divided into 3 groups (Fig 1). Group 1 included the materials from Sichuan except SAG-E17 (which was from Huili) and SAG-E1 (which was from Shanxi). Group 2 consisted of 4 materials from Yunnan, in which one material was collected from Sandu of Guizhou (SAG-E26) while one material was from Nandan of Guangxi (SAG-E29). Group 3 contained the materials from Guizhou (except SAG-E26), Guangxi (except SAG-E29), Guangdong, Hainan, and one material from Huili of Sichuan (SAG-E17). Three subgroups were consisted into group 1, The first subgroup included the materials from the middle reaches of Minjiang River basin (Dujiangyan, Wenjiang, Longquan and Xinjin, except Jiajiang), the second subgroup consisted of the materials from the Qinyi River basin (Ya′an, Shimian and Hanyuan), Meishan city (the middle reaches of Minjiang River basin), and Leshan (the lower reaches of Minjiang River basin), the third subgroup contained the materials from Panxi area (Panzhihua, Huili and Ningnan). In group 3, the materials from Guizhou province except SAG-E26 from Sandu were divided into one subgroup, the materials from Guangxi province except SAG-E29 of Nandan and Guangdong province except SAG-E39 of Xinyi were divided into another subgroup, the materials from Hainan province were divided into the last subgroup.

**Fig 2:**
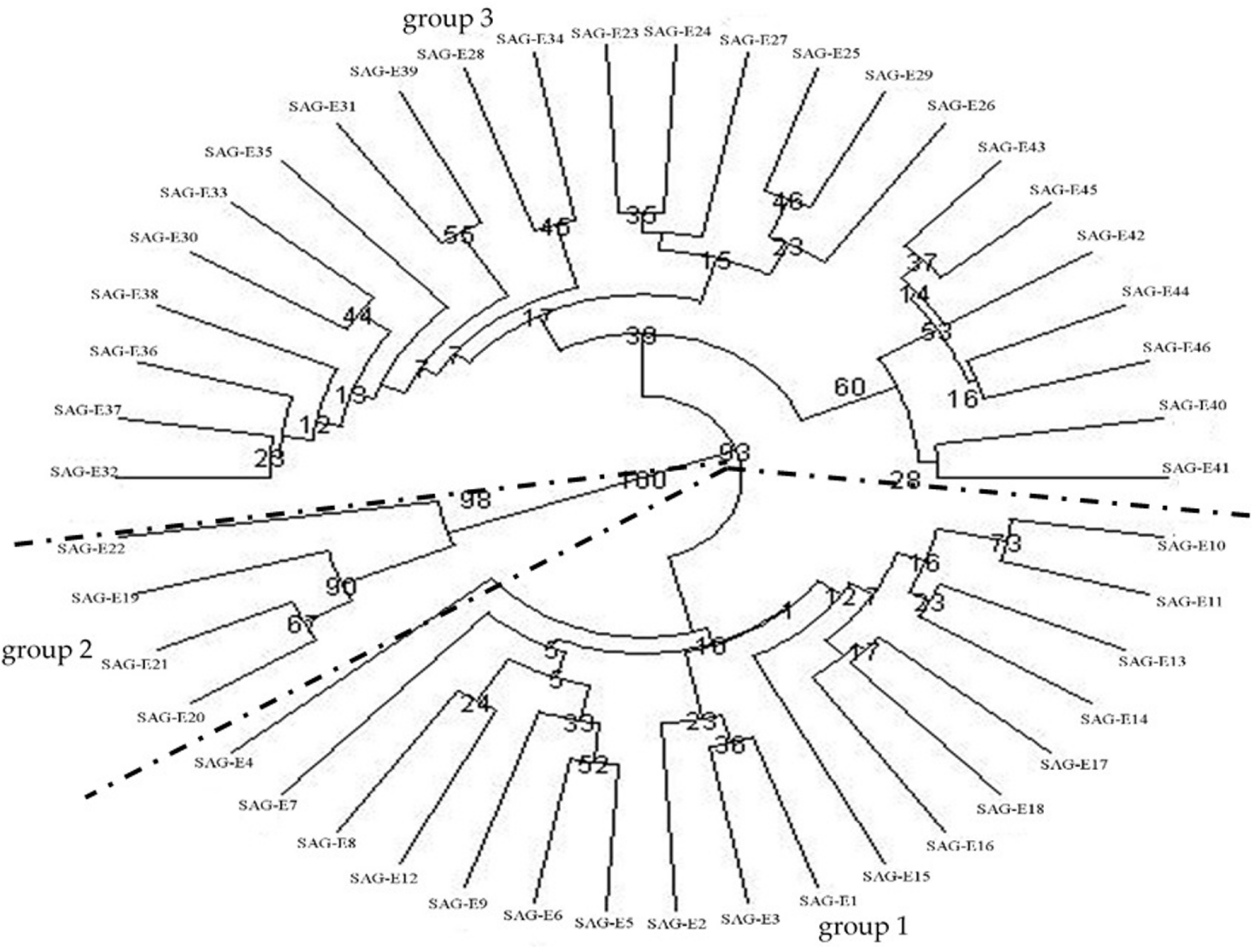
UPGMA cluster analysis based on Nei’s genetic distances of SSR.

For SSRs, the UPGMA tree was composed of 3 major groups from 46 materials which was consistent with the results of ISSRs (Fig 2). Group 1 included the materials from Sichuan province and Shanxi province (SAG-E1). Group 2 consisted of 4 materials from Yunnan province. Group 3 contained the materials from Guizhou, Guangxi, Guangdong, and Hainan, as well as and one material from Sichuan province. In group1, the materials from Hanzhong of Shanxi province, Guangyuan and Suining in Sichuan province were divided into one subgroup, the materials from the middle reaches of Minjiang River basin (Shuangliu, Longquan, Dujiangyan, Xinjin, Wenjiang and Meishan) were divided into one subgroup, the materials from the Qinyi River basin (Ya′an and Shimian) and the lower reaches of Minjiang River basin (Jiajiang and Leshan) were divided into another subgroup, the materials from Panxi area (Panzhihua, Huili and Ningnan) were divided into the last subgroup. In group 3, the materials from Guizhou province and Nandan of Guangxi province were divided into one subgroup, the materials from Guangxi province except SAG-E29 from Nandan and Guangdong province were divided into another subgroup, the materials from Hainan province were divided into the last subgroup, respectively.

The Mantel tests indicated that medium relationship existed between genetic distance and geographic distance among these materials (ISSR: r = 0.520 and p< 0.05, SSR: r = 0.689, p< 0.05). The relationship between SSR gene diversity (HE) values and ISSR HE values was in a medium high level (r = 0.584, p< 0.05).

There were 4 impacts of geographic environments and ploidy level on cluster structure of *E*. *arundinaceus*. The first impact was that the low level of genetic diversity of island *E*. *arundinaceus* due to ocean isolation. Materials with similar geographical distances were gathered into different groups because of the isolation effects of Qiongzhou Strait. For example, E40 (19°05′, 110°10′) was located at a distance of 128.98 km from E38 (20°55′, 110°03′) and 267.34 km from E45 (18°33′, 109°37′) although it was separated from E38 and gathered together with E45. The second impact showed that the low level of genetic diversity of *E*. *arundinaceus* was generated by mountains isolation, which caused materials with similar geographical distances in different groups due to the isolation effects of the mountains. For example, E23 (26°16′, 107°29′) was located at a distance of 470.90 km from E18 (26°58′, 102°48′) and 651.20 km from E38 (20°55′, 110°03′) although it was separated from E18 and gathered together with E38. The third impact was caused by the river systems which was the reason for the materials from the same basin or from different basins gathering together at the river confluence reaches. The fourth impact that the effects of ploidy level on cluster was identified little in this study because 5 tetraploid materials from different geographical locations were not clustered together.

## Discussion

### Effects of the ocean and the big mountains on genetic variation

The percentage of polymorphic bands (PPB) of *E*. *arundinaceus* in this study were 77% (ISSR) and 81% (SSR), lower than 99.3% (AFLP) in the study of Cai [16], higher than 64.9% (ISSR) and 70.1% (RAPD) by Zhang et al [14,15], 69.2% (AFLP) by Tsuruta [24], respectively. These results revealed a high level of genetic diversity for *E*. *arundinaceus* in China. The level of genetic diversity varied among the materials in different regions of China. The materials from Hainan province showed the lowest level of genetic diversity and the value of GS was 0.865 and 0.872 for ISSR and SSR, respectively. The genetic diversity level of *E*. *arundinaceus* in this study was similar with that detected in Cai's study in which was 0.846.

The genetic diversity level of *E*. *arundinaceus* in this study was similar with that detected in Cai's study [16]. These results also supported Zhang's speculation that the low level of genetic diversity of island *E*. *arundinaceus* was generated by ocean isolation, which may have effectively blocked gene flow from germplasm outside the islands, consequently reducing genetic diversity [25].

The “isolation effect” of ocean not only affected the level of genetic diversity, but also the cluster results of materials in different regions. From the cluster analysis of ISSRs and SSRs, the materials from Hainan, Guangxi except Nandan, and Guangdong province were divided into the same subgroup, and the materials from Hainan were separated clearly from others, which showed a large influence of the Qiongzhou Strait on the cluster structure. Similarly, mountains, especially the high mountains, could impact on the cluster results. From the cluster analysis of SSR, the materials were divided into 3 groups. The materials from Sichuan and a material from Hanzhong of Shanxi around Sichuan Basin formed the group 1. Four materials seperated by the Yunnan-Guizhou Plateau from southern tip of Yunnan were clustered in the group 2. The materials from Guizhou, Guangxi, Guangdong and Hainan province formed the group 3. The group 3 was located in the south of Sichuan and in north of the Yunnan, which were just separated by mountains of the Yunnan-Guizhou Plateau. The cluster analysis of ISSRs was consistent with SSRs. Therefore, the ocean and the big mountains could hinder the gene exchange of *E*. *arundinaceus*, and influence the diversity of *E*. *arundinaceus* as well as the genetic relationship among different groups of *E*. *arundinaceus* [26].

### Effect of river systems on genetic variation

The group 1 from cluster analysis of ISSRs was composed of 2 subgroups. The subgroup contained the materials from the middle reaches of Minjiang River basin. The second subgroup included the materials from the Qinyi River basin, one material from the middle reaches of Minjiang River basin, and one material from the lower reaches of Minjiang River basin. For the two subgroups in group 1 SSRs, the materials from the middle reaches of Minjiang River basin were divided into one subgroup, and the materials from the Qinyi River basin and the lower reaches of Minjiang River basin were divided into another subgroup. From the cluster analysis of ISSRs and SSRs, some interesting results were found that the materials from the same river basin were firstly grouped together, while some materials from different river basin were also grouped together, such as the materials from the Qinyi River basin and the lower reaches of Minjiang River basin, which indicated the gene exchange of *E*. *arundinaceus* may be promoted through confluence of river systems. Meanwhile, the materials from near location tended to be grouped together through gene flow, due to *E*. *arundinaceus*'s characteristic of outcrossing with higher level of gene flow [27]. Gene flow of *E*. *arundinaceus* could be blocked by geographical environment (like ocean and mountains). However, the blocked effects of geographical environments on gene flow could also be removed by rivers to provide channels for gene flow, and promote the gene exchange among the materials in the same river basin. Thus, the river systems should be an important factor for the consistent genetic background among the materials from the same river basin.

The mantel tests showed a weak associated relationship existed between genetic distance and geographic distances among these materials. Because the geographical environment has a great influence on the genetic diversity of *E*. *arundinaceus*, which could hinder or promote the gene exchange between different regions. Previously, genetic variation was affected by geographical environments through limited gene exchange [16, 25], which was consistently identified in this study. Because geographical environments could promote the gene exchange, change the genetic diversity and affect cluster structure among materials.

In this study, both ISSRs and SSRs revealed that the native Chinese *E*. *arundinaceus* materials had a high level of genetic diversity although the correlation coefficient between SSR gene diversity (HE) values and ISSR HE values was small (r = 0.584, p< 0.05). For the geographic factors, the results indicated that the ocean and the big mountains could hinder the gene exchange of *E*. *arundinaceus* and the gene exchange of *E*. *arundinaceus* could be promoted through confluence of river systems.

### Effect of ploidy level on genetic variation

Polyploidization is a common phenomenon in plant evolution which is also useful for the formation of new species [28]. Shock of polyploidization in the genome could cause chromosome recombination and sequence elimination which would lead to genetic variation [29–30]. The effects of ploidy level on genetic variation of Chinese *Dactylis glomerata* was studied by Zeng, which showed the ploidy level was the primary factor of the effect on Orchardgrass cluster structure and the genetic variation. And the genetic diversity of among the materials with same ploidy level were smaller than the materials with difference ploidy level [31]. In previous study, we inferred that the altered ploidy might contribute to the genetic variation in the Chinese *E*. *arundinaceus* germplasm since gene flow between plants of altered ploidy is likely limited [25]. However, the cluster structure wasn’t affected by altered ploidy in this study, so ploidy level may have little effect on genetic variation of *E*. *arundinaceus*. The effects of polyploidization on the plant genome may be little in some polyploid species, which led to chromosome recombination and sequence elimination. Moreover, polyploidization were not tested in these species [30].

## Supporting information

S1 Table ISSRs data for 46 *E*. *arundinaceus* materials amplified using 7 markers, coded as presence (1) and absence (0). (XLSX)

S2 Table SSRs data for 46 *E*. *arundinaceus* materials amplified using 15 markers, coded as presence (1) and absence (0). (XLSX)

S3 Table Genetic similarity between E. arundinaceus materials by ISSR markers. (XLSX)

S4 Table Genetic similarity between E. arundinaceus materials by SSR markers. (XLSX)
